# Effects of organic acid, *Enterococcus faecalis* strain EC-12 and sugar cane extract in feed against enterotoxigenic *Escherichia coli*-induced diarrhea in pigs

**DOI:** 10.1186/s13568-021-01228-2

**Published:** 2021-05-13

**Authors:** Hiroki Matsumoto, Masashi Miyagawa, Yizhe Yin, Takayuki Oosumi

**Affiliations:** Research and Development Section, Institute of Animal Health, JA Zen-noh (National Federation of Agricultural Cooperative Associations), 7 Ohja-machi Sakura-shi, Chiba, 285-0043 Japan

**Keywords:** Enterotoxigenic *Escherichia coli*, Gut microbiota, Non-antimicrobial, Organic acid, Pigs, Post-weaning diarrhea

## Abstract

Diarrhea can lead to mortality and delayed growth in pigs and is a major economic loss in the pig industry. In this study, we evaluated non-antimicrobial materials that can prevent diarrhea to infection by enterotoxigenic *Escherichia coli* (ETEC) in weaned pigs and investigated biological changes. We confirmed the efficacy of fumaric acid, lactic acid, *Enterococcus faecalis* strain EC-12 (EC12) and sugar cane extract (SCE) in inhibiting diarrhea and investigated the biological changes by analyzing gut microbiota and plasma metabolites. Administration of EC12 (0.1%, w/w) and SCE (1.0%, w/w) groups had reduced score of diarrhea. Furthermore, the combination of EC12 and SCE was effective at reducing the fecal score of diarrhea even at low concentrations. Administration of either EC12 or SCE greatly reduced the relative abundance of *Enterobacteriaceae* in pigs. EC12 and SCE were most effective in suppressing ETEC-induced diarrhea in weaned pigs. Furthermore, we were able to identify biological changes in pigs when EC12 and SCE were administered to pigs. These feeds may have prevented infection by ETEC in weaned pigs and may improve pig productivity and reduce the use of antimicrobial agents.

## Introduction

Post-weaning diarrhea (PWD) by enterotoxigenic *Escherichia coli* (ETEC) has significant economic impact on swine production worldwide (Amezcua et al. 2002; Li et al. 2020). ETEC that induces PWD produces heat-labile enterotoxin (LT) and/or heat-stable enterotoxin (STa, STb) and are mainly expressed in F4 or F18 fimbriae. Infection with ETEC in pigs causes an inflammatory response in intestinal epithelial cells and induces diarrhea, which leads to growth delay and mortality (Fairbrother et al. 2005). In general, antimicrobials are often used to prevent PWD induced by bacteria such as ETEC, and the increase in the number of antimicrobial resistance (AMR) bacteria has become a problem around the world (Sayan et al. 2018). Therefore, to prevent further increases in AMR bacteria, there has been a worldwide movement to ban supplementation of feed with antimicrobial agents to promote the growth of animals (Casewell et al. 2003; Dębski 2016; Nhung et al. 2016; Luise et al. 2019). New strategies that replace antimicrobials have been investigated in the swine industry to prevent PWD. These strategies include supplementation with zinc oxide, organic acid, probiotics, prebiotics, essential oils and others.

Addition of organic acids in feed reduces the number of pathogenic bacteria in the stomach and small intestine (Kluge et al. 2006). When piglets are given feed supplemented with an organic acid such as citric acid, fumaric acid, lactic acid, or formic acid, the pH of the stomach is lowered, and the bactericidal effect is imparted such that the growth and health of the pigs are improved (Pettigrew 2006; Tsiloyiannis et al. 2001; Edmonds et al. 1985; Giesting and Easter 1985). There are also reports that feeding fumaric acid reduces ETEC-induced diarrhea (Owusu-Asiedu et al. 2003).

Heat killed inactivated and dried cell preparations of *Enterococcus faecalis* strain EC-12 (EC12) (FERM BP-10,284) is one of the biogenics expected to reduce PWD. Oral administration of EC12 as a supplement at 0.05% (w/w) in basal diets prevents edema disease in weaning pigs (Tsukahara et al. 2004). But this supplement can be costly. If a small amount of EC12 can prevent ETEC-induced diarrhea, it can be very useful in weaning pigs.

Sugar cane extract (SCE) is a natural product that can stimulate the immune system and also has anti-inflammatory, antioxidant and anti-stress activity (Ledón et al. 2003; El-Abasy et al. 2002; Takara et al. 2002). SCE is known to have a protective effect against *Eimeria tenella* infection in chickens (El-Abasy et al. 2003). Additionally, the administration of SCE improves immune function and weight gain in pigs (Lo et al. 2006).

The most effective methods of exploring agents that have the potential to prevent PWD is in vivo experimental infection. However, if an inappropriate infection model is used to evaluate the efficacy of these agents, an increased number of pigs may be required for accurate evaluation (Luise et al. 2019). In our previous study, we established high-precision and reproducible experimental infection by controlling factors on the pig-end (MUC4 genotype) and the bacterial-end (adhesion ability) (Matsumoto et al. 2020). In this study, we evaluated the effectiveness of several candidate agents such as organic acids, EC12 and SCE, against PWD using an established animal experimental model.

## Materials and methods

### Ethics statement

This study was approved by the Animal Experimental Review Committee (Ethical Approval Code No. 335, 343, 350 and 401).

### Experimental animals

All animal studies were performed at the JA Zen-noh Institute of Animal Health. A total of 108 male piglets (Landrace) that had been genotyped for *MUC4*. were used. All pigs were derived from specific pathogen-free herds and were transported into the Zen-noh Institute of Animal Health on the day of weaning (21 day of age) (d 0) and were housed in individual pens. Body weights were measured at the beginning of the experiment (d 0) and at autopsy (d 12) or at the time of death. Pigs were fed a basal diet (Table 1) with or without supplements of approximately 400 g per day throughout the test period (d 0 to 12) and were provided water *ad libitum* via a push drinker.

**Table 1:** Ingredients of the basal diet used in this study

Ingredients (%)		Calculated (%)	
Brown rice for feed	24.20	CP	21.81
Whey powder	22.10	Fat	4.81
Soybean meal	16.30	Fibre	1.22
Corn	8.20	Ash	6.48
Wheat	4.80	Ca	0.89
Skim milk powder	4.70	P	0.73
Potato protein	3.00	Lysine	1.63
Fishmeal	4.10	Met + Cys	1.00
Mixed fats and oils	3.20		
Calcium carbonate	0.64		
Salt	0.30		
Choline chloride	0.06		
Calcium phosphate	0.91		
Methionine	0.27		
Lysine	0.26		
Threonine	0.10		
Sugar	4.00		
Other	2.90		

### Experimental designs

First, we performed a screening test to determine which supplements are highly effective in suppressing ETEC-induced diarrhea. The test groups were divided as follows: (1) The control group (CO) was fed basal diet only (10 pigs); (2) the fumaric acid group (FA) was fed basal diet supplemented with 1.0% (w/w) fumaric acid (8 pigs); (3) the lactic acid group (LA) was fed basal diet supplemented with 1.0% (w/w) lactic acid (8 pigs); (4) the *Enterococcus faecalis* strain EC-12 (EC12) (FERM BP-10,284) high-dose group (EH) was fed basal diet supplemented with 0.1% (w/w) EC12 (5 pigs); (5) the EC12 middle dose group (EM) was fed basal diet supplemented with 0.01% (w/w) EC12 (8 pigs); (6) the EC12 low dose group (EL) was fed basal diet supplemented with 0.005% (w/w) EC12 (8 pigs); (7) the sugar cane extract (SCE) high dose group (SH) was fed basal diet supplemented with 1.0% (w/w) SCE (5 pigs); (8) the SCE middle dose group (SM) was fed basal diet supplemented with 0.5% (w/w) SCE (8 pigs); (9) the SCE low dose group (SL) was fed basal diet supplemented with 0.1% (w/w) SCE (8 pigs).

Second, we tested a combination of supplements (Mix; mixture of EC12 and SCE) that were highly effective in inhibiting ETEC-induced diarrhea. The test groups were distributed as follows: (1) The control group (CO) was fed basal diet only (8 pigs); (2) the mix high dose group (MH) was fed basal diet supplemented with 0.005% (w/w) EC12 and 0.25% (w/w) SCE (8 pigs); (3) the mix low dose group (ML) was fed basal diet supplemented with 0.001% (w/w) EC12 and 0.05% (w/w) SCE (8 pigs).

Third, the biological activity of EC12 and SCE and their mixtures was confirmed. The test groups were distributed as follows: (1) The control group (CO) was fed basal diet only (4 pigs); (2) the EC12 group (EC) was fed basal diet supplemented with 0.1% (w/w) EC12 (4 pigs); (3) the SCE group (SC) was fed basal diet supplemented with 1.0% (w/w) SCE (4 pigs); (4) the mix group (MI) was fed basal diet supplemented with 0.005% (w/w) EC12 and 0.25% (w/w) SCE (4 pigs). In the first and second tests, all pigs were challenged with ETEC. Table 2 is a summary of all of the animal experiments.

**Table 2:** Animal experimental design (treatment, number of animals, ETEC infection) in this study

	Group	Treatment	n =	F4 ETEC challenged
First	CO	Only basal diet	10	+
	FA	Basal diet with fumaric acid (1.0%, w/w)	8	+
	LA	Basal diet with lactic acid (1.0%, w/w)	8	+
	EH	Basal diet with EC12 (0.1%, w/w)	5	+
	EM	Basal diet with EC12 (0.01%, w/w )	8	+
	EL	Basal diet with EC12 (0.005%, w/w)	8	+
	SH	Basal diet with SCE (1.0%, w/w)	5	+
	SM	Basal diet with SCE (0.5%, w/w)	8	+
	SL	Basal diet with SCE (0.1%, w/w)	8	+
Second	CO	Only basal diet	8	+
	MH	Basal diet with EC12 (0.005%, w/w) and SCE (0.25%, w/w)	8	+
	ML	Basal diet with EC12 (0.001%, w /w) and SCE (0.05%, w/w)	8	+
Third	CO	Only basal diet	4	–
	EC	Basal diet with EC12 (0.1%, w/w)	4	–
	SC	Basal diet with SCE (1.0%, w/w)	4	–
	MI	Basal diet with EC12 (0.005%, w/w ) and SCE (0.25%, w/w)	4	–

### Experimental infection

We used the non-common *E. coli* (F4:LT:STa:STb) strain. This ETEC strain was grown in LB medium for 6 h at 37 ℃, harvested by centrifugation and were resuspended in 10% skim milk. The pigs were orally challenged by ETEC with a single dose of approximately 10^9^ CFU per pig at 26 days of age (d 5). At d 12 in the first and second animal experiments (screening and mixture evaluation), pigs were euthanized using sodium pentobarbital under sedation with xylazine and midazolam for all experiments.

### Evaluation of diarrhea

The feces of the pigs were examined every morning. Fecal properties were measured as follows: 0 (normal), 1 (soft feces), 2 (semi-liquid) and 3 (liquid). The incidence of diarrhea refers to the percentage of animals with a score of 1 or more in each group, except for pigs that died at 1 days post infection (dpi). The duration of diarrhea was recorded individually in pigs that survived to the end (d 12), and the mean duration for the group was calculated. The mortality rate was recorded throughout the monitoring period.

### Microbiota analysis for 16 S rRNA V3-V4

Bacterial DNA was extracted from the intestinal contents of non-challenged pigs in the third animal experiment using the All Prep PowerViral DNA/RNA kit (QIAGEN, Tokyo, Japan) according to the manufacturer’s instructions, followed by measurement of the DNA concentration and purity using a NanoDrop (Thermo Scientific Scientific, Tokyo, Japan). The extracted DNA was kept at − 20 ℃ until used for 16 S rRNA PCR. The V3–V4 region of the bacterial 16 S rRNA gene was amplified by following the Illumina 16 S Metagenomic Sequencing Library Preparation guide. Final DNA concentrations of the purified products were measured with a Qubit 3.0 fluorometer (Thermo Fisher Scientific, Tokyo, Japan). The purified products were mixed in equal molar. The 16 S rRNA gene libraries were sequenced with 2 × 300 bp paired-end reads on the MiSeq systems (Illumina, Tokyo, Japan), using MiSeq v3 reagent kits (Illumina, Tokyo, Japan) (BioProject PRJDB11197). Sequences were grouped as Operational Taxonomic Units (OTUs), assigned to taxonomic groups using the Greengenes database, and clustered with the help of CLC Microbial Genomics Module software(QIAGEN, Tokyo, Japan). An analysis of Chao1 (alpha) diversity index was performed with the CLC Microbial Genomics Module.

### Plasma metabolome analysis

Plasma samples of non-challenged pigs in the third animal experiment were stored at − 80 ℃ until measured. Metabolite extraction and metabolome analysis were conducted at Human Metabolome Technologies Inc. (HMT, Yamagata, Japan). Metabolome analysis was conducted using a Basic Scan package at HMT using CE-TOFMS. The analysis was carried out using an Agilent CE system (Agilent Technologies, Palo Alto, CA, USA) equipped with an Agilent 6210 TOFMS (Agilent Technologies) at a service facility at HMT.

### Statistical analysis

The mean fecal score was analyzed with repeated-measures ANOVA, with dpi as a within subject factor and the test groups as the between-subject factor. The data was adjusted for multiple comparisons using the Dunnett test against the CO group. Pigs with a fecal score of 1 or more during the experimental period were considered to have diarrhea. The incidence of diarrhea and mortality were analyzed using Fisher’s exact test separately against the CO group. Body weights, duration of diarrhea, alfa diversity (Chao1) and relative abundance of microbiota were analyzed with single factor ANOVA after the Bartlett test and multiple comparisons by the Dunnett test against the CO group. Metabolites were analyzed with Welch’s t test separately against the CO group. Statistical analyses were performed using Excel 2016 (Microsoft Corporation) with the add-in software Statcel 4. A p-value of less than 0.05 was considered to be significantly different between groups.

## Results

### Screening test to identify substances that are highly effective in suppressing diarrhea caused by ETEC in weaning pigs

The occurrence of diarrhea was confirmed in all pigs, and the mean fecal score in the CO group fed only the basal diet (1.72) was the highest among all test groups (Table 3). Diarrhea was induced in the FA, LA group fed basal diet mixed with organic acid, but the mean fecal scores in the FA and LA groups (1.09 and 0.91, respectively) were significantly lower than that in the CO group. Addition of EC12 in the basal diet in the different dosage groups (EH, EM and EL) resulted in a significant reduction in the mean fecal score (0.17, 0.66 and 0.78, respectively). Furthermore, the incidence of diarrhea (40.0 %), duration of diarrhea (1.0 day) and mortality (0 %) were also significantly improved in the EH group compared to the CO group. Pigs fed SCE mixed with the basic diet showed a significant reduction in the mean fecal score (respectively 0.31 and 0.86) in the SH and SL group. There was also a significant improvement in mortality (0 %), incidence of diarrhea (80.0 %), and duration of diarrhea (1.8 day) in the SH group compared to the CO group.

The initial body weight was not different from that of the CO group in other test groups, but the final body weight of the EH, EM, SH and SL groups was significantly higher than that of the CO group.

**Table 3:** Effect of diet supplementation against PWD in weaning pigs

	Treatment^a^										SEM
	CO	FA	LA	EH	EM	EL	SH	SM	SL		
	n = 10	8	8	5	8	8	5	8	8		
Mean body weight (kg)											
Initial	5.35	5.19	5.63	5.60	6.00	4.88	6.30	6.13	5.72		0.12
Final^b^	5.30	6.69	6.50	7.50*	8.13*	6.75	7.70*	6.75	7.13*		0.18
Mean fecal score	1.72	1.09*	0.91*	0.17*	0.66*	0.78*	0.31*	1.30	0.86*		0.05
(1–7 dpi)^c^											
Diarrhea incidence (%)	100	100	100	40.0*	75.0	85.7	80.0	75.0	100		
Duration of diarrhea (day)	5.0	3.8	3.5	1.0*	3.0	2.7	1.8	3.6	3.3		0.29
Mortality (%)	60.0	25.0	25.0	0*	25.0	12.5	0*	12.5	37.5		

^a^CO (Control); basal diet only, FA; with fumaric acid (1.0 %, w /w), LA; with lactic acid (1.0 %, w /w), EH; with EC12 high dose (0.1 %, w /w), EM; with EC12 middle dose (0.01 %, w /w), EL; with EC12 low dose (0.005 %, w /w), SH; with SCE high dose (1.0 %, w /w), SM; with SCE middle dose (0.5 %, w /w), SL; with SCE low dose (0.1 %, w /w)

^b^Measured at necropsy (d 12) or death

^c^Indicate d 6 to d 12. *Means within rows are significantly different (P < 0.05 vs. CO group)

### Effect of EC12 and SCE at low concentrations reduced ETEC-induced diarrhea

The mean fecal score (1.63), diarrhea incidence (100 %), duration of diarrhea (4.5), and mortality (50.0 %) in the CO group fed only the basic diet were comparable to those in the secondanimal experiment (Table 4). The combination of EC12 and SCE supplements mixed with the basic diet in both MH (EC12; 0.005 % (w /w) and SCE; 0.25 %( w /w)) and ML (EC12; 0.001 % (w /w) and SCE; 0.05 %( w /w)) groups resulted in a significant decrease in the mean fecal score (0.47 and 0.94, respectively). The initial body weight was not different from that of the CO group in other test groups, but the final body weights of the MH and ML groups were significantly higher (7.50 and 6.69 kg, respectively) than that of the CO group (5.19 kg).

**Table 4:** Effect of diet mixed with EC12 and SCE against PWD in weaning pigs

	Treatment^a^			SEM
	CO	MH	ML	
	n = 8	8	8	
Mean body weight (kg)				
Initial	5.06	5.31	5.38	0.23
Final^b^	5.19	7.50*	6.69*	0.26
Mean fecal score	1.63	0.47*	0.94*	0.10
(1–7 dpi)^c^				
Diarrhea incidence (%)	100	71.4	100	
Duration of diarrhea (day)	4.5	2.3	4.0	0.49
Mortality (%)	50.0	14.3	14.3	

^a^CO (Control); basal diet only, MH (Mix high dose); with EC12 (0.005 %, w /w) and SCE (0.25 %, w /w), ML (Mix low dose); with EC12 (0.001 %, w /w) and SCE (0.05 %, w /w). ^b^Measured at necropsy (d 12) or death. ^c^Indicate d 6 to d 12. *Means within rows are significantly different (P < 0.05 vs. CO group)

### **Microbiota profile in the jejunum, ileum and cecum*via*16 S rRNA sequence analysis**

Microbiota was examined in the EC12 and SCE groups or in combination with EC12 and SCE (MI group) without ETEC infection. Bacterial community compositions in the jejunum, ileum and cecum were targeted by amplifying the V3–V4 region of the 16 S rRNA gene. After quality filtering, 7,588,426 clean reads were obtained with an average of 161,456 assigned to 2,361 different operational taxonomic units (OTUs).

In the jejunum and ileum, a significant increase in alpha diversity (Chao 1 index) was found in all treatment groups compared to the CO group (Table 5). There were no significant changes in the cecum.

**Table 5:** Chao1 (alpha) diversity index of intestinal microbiota with administration of EC12, SCE, and mixture of EC12 and SCE in weaning pigs without ETEC infection

	Treatment^a^				SEM
	CO	EC	SC	MI	
Jejunum	153.6	337.5*	379.0*	253.1*	25.1
Ileum	140.4	300.4*	420.5*	452.7*	32.4
Cecum	729.7	698.0*	677.9*	703.3*	14.2

^a^CO (Control); basal diet only, EC; with EC12 (0.1 %, w /w),SC; with SCE (1.0 %, w /w), MI (Mix); with EC12 (0.005 %, w /w) and SCE (0.25 %, w /w). * Means within rows are significantly different (P < 0.05 vs. CO group)

In the CO group fed the basic diet only, the most predominant microbiota in the jejunum and ileum were *Enterobacteriaceae* (jejunum; 71.25 %, ileum; 51.75 %) (Table 6). On the other hand, the relative abundance of *Enterobacteriaceae* in the EC, SC and MI groups was significantly lower than in the CO group in both the jejunum (8.75 %, 13.50 and 15.00 %, respectively) and ileum (6.75 %, 34.25 and 4.00 %, respectively). In the CO group, *Clostridiaceae* was rarely detected, but in the EC group, an increase in the relative abundance of *Clostridiaceae* was found in both the jejunum (CO; 0.18 %, EC; 36.33 %) and ileum (CO; 0.31 %, EC; 6.15 %). In the CO group, the relative abundance of *Lachnospiraceae* was low, but in the SC (1.50 %) and MI (3.25 %) groups, *Lachnospiraceae* was increased in the ileum. An increase in the relative abundance of *Lactobacillaceae* was found in the jejunum (5.37 %) and ileum (3.34 %) of the EC group and in the jejunum (6.81 %) of the SC group. The relative abundance of *Lactobacillaceae* in the MI group was slightly higher than in the CO group in both the jejunum (CO; 0.03 %, MI; 2.32 %) and ileum (CO; 0.04 %, MI; 0.24 %), but the results were not significantly different. *Prevotellaceae*, *Ruminococcaceae*, *Lachnospiraceae*, *Lactobacillaceae*, and *Paraprevotellaceae* were predominant in the cecum, with no significant differences among any test group.

**Table 6:** Relative abundance of intestinal microbiota with administration of EC12, SCE, and mixture of EC12 and SCE in weaning pigs without ETEC infection

Family	Treatment^a^				
	CO	EC	SC	MI	SEM
Jejunum					
* Enterobacteriaceae*	71.25	8.75^*^	13.50^*^	15.00^*^	6.67
* Pasteurellaceae*	22.25	22.25	21.75	13.90	4.70
* Clostridiaceae*	0.18	36.33^*^	1.15	0.05^*^	4.83
* Lactobacillaceae*	0.03	5.37^*^	6.81^*^	2.32	0.92
* Weeksellaceae*	0.00	0.55	15.06^*^	0.01	2.00
Ileum					
* Enterobacteriaceae*	51.75	6.75^*^	34.25^*^	4.00^*^	5.12
* Pasteurellaceae*	40.75	65.00^*^	43.75	54.25^*^	2.89
* Lachnospiraceae*	0.01	0.18	1.50^*^	3.25^*^	0.35
* Clostridiaceae*	0.31	6.15^*^	0.43	2.00	0.74
* Lactobacillaceae*	0.04	3.34^*^	0.22	0.24	0.42
Cecum					
* Prevotellaceae*	17.75	18.75	17.25	17.75	1.20
* Ruminococcaceae*	15.25	15.25	17.75	16.00	0.95
* Lachnospiraceae*	11.25	11.25	13.00	13.75	0.84
* Lactobacillaceae*	11.25	9.00	7.00	10.75	1.94
* Paraprevotellaceae*	10.50	8.75	9.75	9.75	1.35

^a^CO (Control); basal diet only, EC; with EC12 (0.1 %, w /w),SC; with SCE (1.0 %, w /w), MI (Mix); with EC12 (0.005 %, w /w) and SCE (0.25 %, w /w). Data are presented as mean of relative abundance (%)

*Means within rows are significantly different (P < 0.05 vs. CO group)

### Comparison of plasma metabolites

CE-TOFMS of pig plasma revealed 239 metabolites (170 cations and 69 anions). We comprehensively compared the relative levels of metabolites among the EC, SC, MI and CO groups (Table 7). The ratio of butyric acid was significantly higher in the EC group (1.76 times) than in the CO group, and a higher trend was observed in the MI group (1.76 times, *P* = 0.06) as well. There was a trend for high lactic acid levels in all treatment groups, and the ratio was significantly increased in the MI group (2.24 times) in particular. Citric acid was significantly higher in the EC (1.59 times) and MI groups (1.38 times), and isocitric acid was also elevated only in the EC group (1.68 times). Methionine sulfoxide showed a significant decrease in all treatment groups compared to the CO group (EC; 0.39, SC; 0.27, MI; 0.21 times). On the other hand, an increase in the ratio of *N*, *N*-dimethylglycine was observed in all treatment groups (EC; 2.84, SC; 2.13, MI; 2.52 times).

**Table 7:** Ratio of each treatment against the CO (control) group on plasma metabolites with administration of EC12, SCE, and mixture of EC12 and SCE in weaning pigs without ETEC infection

Compound name	Ratio (Treatment^a^/ CO)			*P* value		
	EC	SC	MI	EC vs. CO	SC vs. CO	MI vs. CO
Butyric acid	1.76	1.52	1.76	**0.02**	0.22	0.06
Lactic acid	1.81	1.91	2.24	0.09	0.13	**0.01**
Succinic acid	0.93	1.03	1.15	0.83	0.91	0.64
Pyruvic acid	1.02	1.30	1.33	0.93	0.24	0.28
Malic acid	1.92	1.45	1.57	0.12	0.15	0.26
Citric acid	1.59	1.03	1.38	**0.02**	0.81	**0.02**
Isocitric acid	1.68	0.99	1.38	**0.03**	0.92	0.06
Methionine sulfoxide	0.39	0.27	0.21	**0.01**	**< 0.01**	**0.01**
Carnosine	1.40	0.93	1.05	0.33	0.82	0.83
* N*,*N*-Dimethylglycine	2.84	2.13	2.52	**0.01**	**0.02**	**0.04**
5-Oxoproline	1.38	1.13	1.25	**0.04**	0.26	0.27
Creatinine	1.25	0.97	1.15	**0.04**	0.73	0.10
Phosphocreatine	4.31	1.58	1.43	0.29	0.05	0.44
Urea	0.79	0.65	0.50	0.26	**0.02**	**< 0.01**
2-Oxoglutaric acid	1.53	1.46	1.68	**0.04**	0.14	**0.04**
Carnitine	1.34	1.15	1.09	0.20	0.50	0.64
Cholic acid	2.14	0.54	2.17	0.48	**0.03**	0.16
Choline	1.07	0.96	0.85	0.68	0.76	0.31
Glycocholic acid	0.65	4.68	0.59	0.46	0.53	0.41
7-Methylguanine	1.04	0.81	0.93	0.77	0.27	0.47
*N*^6^-Acetyllysine	1.39	0.92	1.00	**0.01**	0.63	0.98

^a^CO (Control); basal diet only, EC; with EC12 (0.1%, w/w),SC; with SCE (1.0%, w/w), MI (Mix); with EC12 (0.005%, w/w) and SCE (0.25%, w/w)

## Discussion

Feeding fumaric acid or lactic acid mixed with the basal diet resulted in significantly lower fecal scores compared to the CO group fed only the basal diet. This finding is consistent with previous reports (Owusu-Asiedu et al. 2003). In this study, however, there was no difference in the body weights compared to the CO group (Table 3). Weight loss due to diarrhea has been shown to affect subsequent development, so it is important to keep these factors in mind (Fairbrother et al. 2005). Based on these results, we could not conclude that organic acids have a sufficient preventive effect on diseases caused by ETEC. Low fecal scores and maintenance of body weight were observed in the group fed with EC12 or SCE mixed with the basic diet compared to the CO group. In particular, high-dose EC12 (EH) improved body weight, fecal score, incidence of diarrhea, duration of diarrhea, and mortality, whereas low-dose EC12 (EL) showed only a slight improvement in fecal score reduction. To increase the potential of EC12 in preventing PWD, we considered combining SCE, which had a smaller but significant effect compared to EC12. This finding is due to the fact that EC12 is approximately 10 times more expensive than other organic acids and SCE.

A second animal study showed that the diarrhea caused by ETEC was suppressed, and the body weight was maintained even at low concentrations of EC12 (Table 4). Previous reports have shown that feeding EC12 at 0.05 % (w /w) or higher could prevent edematous disease (Tsukahara et al. 2004). Our results showed that 0.001% (w/w) of EC12 was effective in preventing PWD and an additive effect was found after simultaneously feeding SCE.

To examine biological changes by feeding of EC12 and SCE to weaned pigs, we analyzed the gut microbiota and measured plasma metabolites (Tables 5, 6 and 7). The most dramatic changes occurred in the relative abundance of *Enterobacteriaceae* in the jejunum and ileum when EC12 and SCE were mixed in the basic diet (Table 6). SCE contains polyphenols and other substances, and it has been reported that these affect the intestinal microbiota (Williams et al. 2017). In this study, it is estimated that the change in the intestinal microbiota even when SCE was fed alone was due to polyphenols, but further studies are needed to clarify these findings. In general, *Enterobacteriaceae* are known to be early colonizers among the bacteria that make up the intestinal microbiota (Hong et al. 2010; Favier et al. 2002). Among the diverse groups of bacteria present in the gut, an increase in *Enterobacteriaceae* has been proposed as a non-invasive biomarker of gut health because they are associated with gut dysbiosis in weaned pigs (Gresse et al. 2017; Eeckhaut et al. 2016; Hughes et al. 2017; Ducatelle et al. 2018). In a healthy gut environment, commensal bacteria are also known to inhibit the establishment and growth of pathogenic microorganisms, a phenomenon known as “colonization resistance” (Kim et al. 2017). The addition of EC12 and SCE may optimize the intestinal environment and, therefore, restrict ETEC infection. In addition, feeding EC12 and SCE increased the relative abundance of *Clostridiaceae* and *Lachnospiraceae* (Table 6). These families contain many bacteria that producebutyric acid, a short-chain fatty acid (Suksong et al. 2019; Cibis et al. 2016). In fact, plasma butyric acidlevels were significantly higher in the EC group compared to the CO group, and there was a trend for higher levels in the SC and MI groups compared to the CO group. Short-chain fatty acids not only lower the pH of the intestine but also inhibit the establishment of pathogenic bacteria by stimulating host immune cells (Caballero and Pamer 2015). Addition of butyric acid to Caco-2 cells was shown to increase transepithelial electrical resistance; thus, butyric acid strengthens barrier functions (Donohoe et al. 2011).

Plasma organic acids such as lactic acid, malic acid and citric acid were slightly higher in the EC and MI group compared to the CO group (Table 7). This may have caused a decrease in pH in the small intestine, which could play an important part in protecting against ETEC infection. Interestingly, all treatment groups showed a decrease in methionine sulfoxide and an increase in *N,N*-dimethylglycine (DMG) as compared to the CO group (Table 7). Among amino acids, methionine residues are the most susceptible to oxidation and quickly respond to intracellular oxidative stress to form sulfoxide. Many cells respond to this by expressing enzymes for reductive repair, but elevated levels of methionine sulfoxide have been observed in certain proteins in the tissues of aging animals. This finding is likely to be involved in the decline in cell function during aging (Martínez et al. 2017; Moskovitz 2005). DMG may improve immune responses in laboratory animals and humans, physical and mental performance in athletes and the elderly, and cardiovascular function in clinical patients (Graber et al. 1981). Other studies indicate that DMG can protect the liver, aid in detoxification, reduce seizure activity in some people, and promote improvement in children and adults with autism (Reap and Lawson 1990). Specifically, with respect to immunomodulation, it has been reported that DMG enhances B and T cell function and cytokine regulation (Lawson et al. 2007). This suggests that feeding EC12 and SCE may reduce oxidative stress and improve immunity.

In conclusion, among the non-antimicrobial substances evaluated in this study, EC12 and SCE were most effective in suppressing ETEC-induced diarrhea in weaned pigs. Furthermore, a combination of EC12 and SCE suppressed diarrhea and induced weight gain even at lower feed concentrations of both substances. This finding could have significant cost benefits. In addition, feeding this mixture induced changes in the gut microbiota, such as a decrease in the relative abundance of *Enterobacteriaceae* and an increase in the relative abundance of *Clostridiaceae* and *Lachnospiraceae* producing butyric acid. This feed mixture also led to an increase in plasma metabolites such as butyric acid, lactic acid, citric acid, *N, N*- dimethylglycine and acetyllysine, a decrease in methionine sulfoxide. These changes may have prevented infection by ETEC in weaned pigs and may improve pig productivity and reduce the use of antimicrobial agents.

## Data Availability

All data generated or analyzed during this study are available from the corresponding author upon reasonable request.

## Abbreviations

AMR: Antimicrobial resistance
DMG: *N,N*-dimethylglycine
ETEC: Enterotoxigenic *Escherichia coli*
LT: Heat-labile enterotoxin
OTUs: Operational Taxonomic Units
PWD: Post-weaning diarrhea
SNP: Single nucleotide polymorphism
ST: Heat-stable enterotoxin
